# Regional homogeneity abnormalities of resting state brain activities in children with growth hormone deficiency

**DOI:** 10.1038/s41598-020-79475-9

**Published:** 2021-01-11

**Authors:** Fanyu Zhang, Bo Hua, Mei Wang, Tengfei Wang, Zhongxiang Ding, Ju-Rong Ding

**Affiliations:** 1 Artificial Intelligence Key Laboratory of Sichuan Province, Sichuan University of Science and Engineering, Zigong, China; 2 School of Automation and Information Engineering, Sichuan University of Science and Engineering, Zigong, China; 3 Department of Radiology, Affiliated Hangzhou First People’s Hospital, Zhejiang University School of Medicine, Hangzhou, China

**Keywords:** Neurology, Biomedical engineering, Neuroscience, Cognitive neuroscience

## Abstract

Growth hormone deficiency (GHD) is a common developmental disorder in children characterized by low levels of growth hormone secretion, short stature, and multiple cognitive and behavioral problems, including hyperactivity, anxiety, and depression. However, the pathophysiology of this disorder remains unclear. In order to investigate abnormalities of brain functioning in children with GHD, we preformed functional magnetic resonance imaging and regional homogeneity (ReHo) analysis in 26 children with GHD and 15 age- and sex-matched healthy controls (HCs) in a resting state. Compared with HCs, children with GHD exhibited increased ReHo in the left putamen and decreased ReHo in the right precentral gyrus, reflecting a dysfunction of inhibitory control. Decreased ReHo was also identified in the orbital parts of the bilateral superior frontal gyrus and the medial part of the left superior frontal gyrus, a finding that correlated with the inappropriate anxiety and depression that are observed in this patient population. Our results provide imaging evidence of potential pathophysiologic mechanisms for the cognitive and behavioral abnormalities of children with GHD.

## Introduction

Short stature, a prevalent disease in the pediatric endocrine clinic, is characterized by an annual growth in height of less than 5 cm and a height greater than two standard deviations below the average for normal children of the same race, sex, and age^1,2^. Short stature may either be idiopathic or caused by growth hormone deficiency (GHD). Idiopathic short stature (ISS) is the most common type of short stature, which refers to short stature without underlying pathological causes^3^. GHD is one of the most common causes of pathological short stature^4^. Studies using clinical psychological evaluations have shown greater social, thought, and attention problems, and greater delinquent behavior in children with GHD than in healthy controls (HCs)^5^. In addition, a large number of magnetic resonance imaging (MRI) studies have shown that patients with GHD have structural abnormalities in the brain, including an interrupted pituitary stalk and a hypoplastic hypophysis^6,7^, as well as an ectopically located posterior pituitary gland^8^.

Functional magnetic resonance imaging (fMRI) reflects changes in activity intensity in brain regions by detecting changes in the amount of oxygen carried by hemoglobin^9,10^ As a result, the fMRI signal is often referred to as the blood oxygen level-dependent (BOLD) signal^11^. Recently, resting-state fMRI (rs-fMRI) techniques have been used widely in studies of brain activity in various neuropsychiatric disorders, including attention-deficit/hyperactivity disorder^12^, social anxiety disorder^13^, mesial temporal lobe epilepsy^14^, and major depressive disorder^15^. An existing rs-fMRI study has shown that compared with children with ISS, children with GHD have lower functional connection density in the left postcentral gyrus, right precentral gyrus, and Brodmann's area of left cerebellar lobules 7b and 6, as well as lower functional connectivity of the brain network involving the posterior cerebellar lobes and sensorimotor network^16^. However, at present, there is no information about the activity within the brain clusters of children with GHD, especially regarding the consistency of activities.

Regional homogeneity (ReHo)^17^ is a method to measure the similarity of a voxel’s activity-time series within a given cluster. It assumes the spontaneous BOLD signal’s change of voxels within clusters are synchronized and can be modulated during cognitive tasks; therefore, it is suitable for analyzing rs-fMRI data^17^. Abnormalities of ReHo may reflect disorders of local brain functioning^18^ and may indicate abnormal mental activity and cognition^19^. This method has been used in the study of many diseases, including epilepsy^20^, attention-deficit/hyperactivity disorder ^21^, and Alzheimer’s disease^18,22^.

Little is known about the changes in spontaneous brain activity, especially the intra-cluster homogeneity of children with GHD in the resting state. In the present study, we performed rs-fMRI and ReHo analysis on children with GHD and HCs. Based on the existing resting-state neuroimaging studies and clinical behavioral studies of children with GHD, we hypothesized that ReHo analysis would identify differences of activity homogeneity in certain clusters between participates with GHD and HCs, especially in brain regions related to social, thought, and behavior problems^5,16^. In addition, we aimed to explore the difference between the two groups on the brain function and provide imaging data for studying the pathophysiological mechanism of abnormal cognitive and behavioral of children with GHD.

## Results

### ReHo for each group

**Figure 1:**
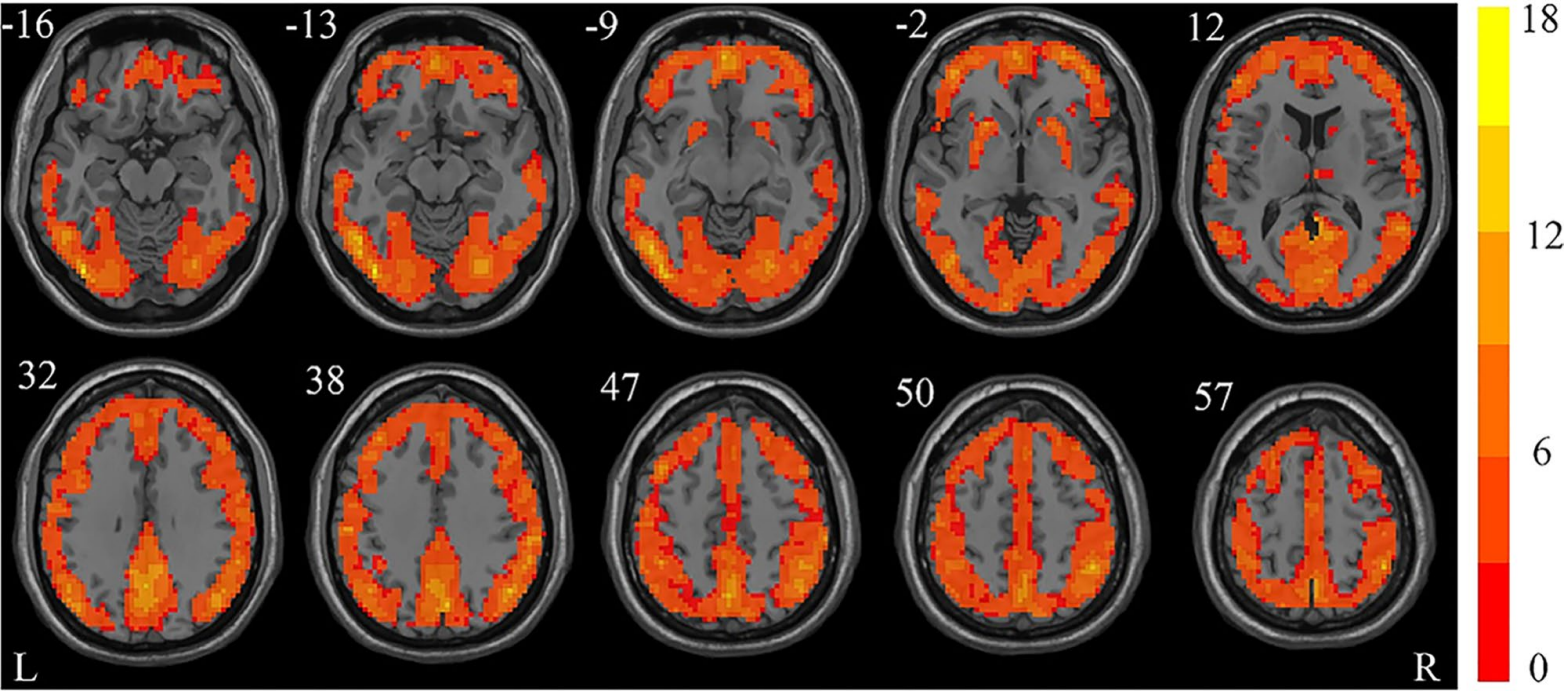
The one-sample *t*-test results of the healthy control group shown as a Kendall’s coefficient concordance (KCC) map (false discovery rate [FDR] corrected, *p* < 0.05). The figure was presented using the free DPABI software. Red areas indicate a positive ReHo. The number above each image refer to the z-plane coordinates of the Montreal Neurological Institute space. *ReHo* regional homogeneity, *L* left, *R* right.

**Figure 2:**
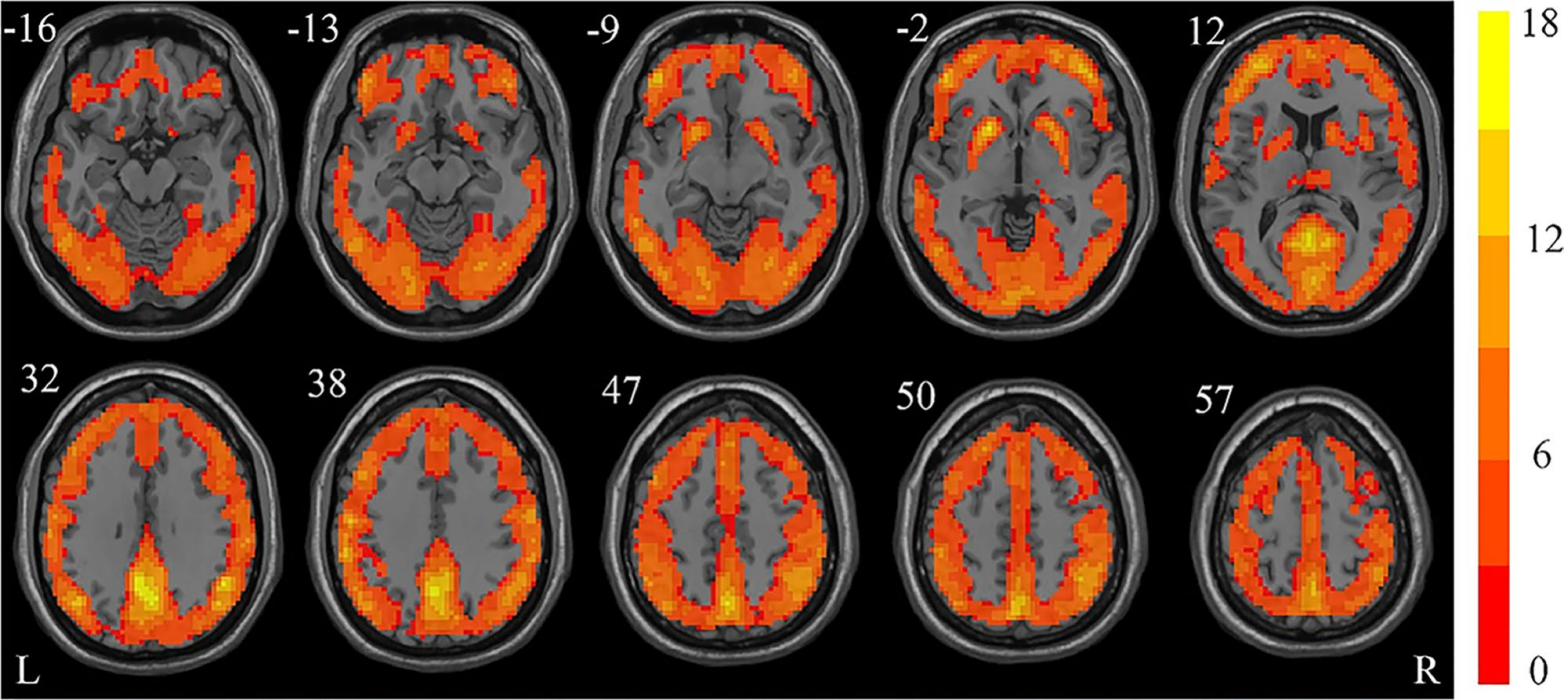
The one-sample *t*-test results of the growth hormone deficiency group shown as a KCC map (FDR corrected, *p* < 0.05). The figure was presented using the free DPABI software. Red areas indicate a positive ReHo. The number above each image refer to the z-plane coordinates of the Montreal Neurological Institute space. *ReHo* regional homogeneity, *L* left, *R* right.

In order to intuitively display ReHo results for the GHD and HC groups, a ReHo map was calculated within each group and graphically displayed in Figs. 1 and 2, respectively (one-sample *t*-test; *p* < 0.05, FDR corrected). Through visual observation, it was found that the ReHo values of the bilateral frontal gyrus, supplementary motor area (SMA), cingulate gyrus, cuneus (CUN), occipital gyrus, fusiform gyrus (FFG), parietal gyrus, angular gyrus (ANG), precuneus (PCUN), putamen (PUT) and temporal gyrus were significantly higher than those of other brain regions. This is consistent with previous research results^13,23^. In addition, the bilateral precentral gyrus (PreCG), calcarine fissure and surrounding cortex^6^, lingual gyrus (LING), postcentral gyrus (PoCG) and supramarginal gyrus (SMG) also exhibited higher ReHo values.

### Group comparison results

**Figure 3:**
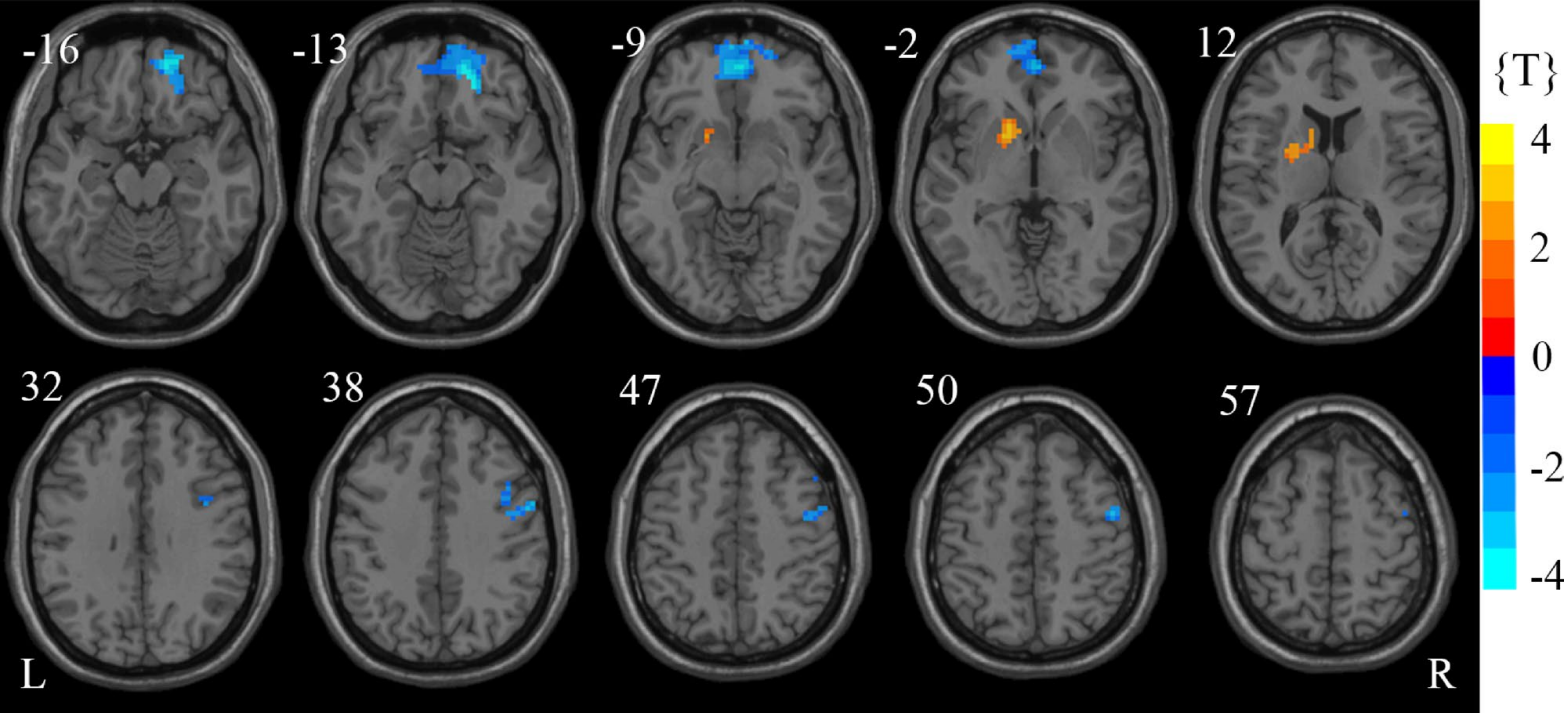
Statistically significant differences in regional homogeneity (ReHo) between children with GHD and healthy controls (*p* < 0.05, AlphaSim corrected). The figure was presented using the free DPABI software. Cold and hot colors in the color bar indicate decreased and increased ReHo, respectively, in children with GHD. The number above each image refer to the z-plane coordinates of the Montreal Neurological Institute space. *GHD* growth hormone deficiency, *L* left, *R* right.

The two-sample *t*-tests results were graphically displayed in Fig. 3. The statistical significance threshold was set at *p* < 0.05, corrected by AlphaSim program (combined a voxel threshold of *p* < 0.05 and a minimum cluster size of 56 voxels)^24^. There were several regions showing significant differences in ReHo between the children with GHD and HCs (Table 1). The left PUT showed increased ReHo, while the right PreCG, bilateral orbital parts of the superior frontal gyrus (ORBsup) and left medial part of the superior frontal gyrus (SFGmed) showed decreased ReHo in children with GHD.

**Table 1:** Regions of increased/decreased ReHo in children with GHD.

Brain region	Voxels	MNI (x,y,z)	*T*	BA
**ReHo increased regions**				
PUT.L	40	− 15, 12, − 3	2.8651	48
**ReHo decreased regions**				
PreCG.R	66	54, 0, 42	− 3.3343	6
ORBsup.R	52	21, 42, − 12	− 4.0408	11
ORBsupmed.L	105	0, 51, − 6	− 3.4678	10/11
ORBsupmed.R	59	3, 51, − 6	− 3.4439	10/11
SFGmed.L	49	0, 36, 57	− 3.1438	8/32

*ReHo* regional homogeneity, *GHD* growth hormone deficiency, *MNI (x,y,z)* coordinates of the peak voxel in the MNI space, *t* the statistical value of peak voxel, with a positive *t-*value indicating an increased ReHo and a negative *t-*value indicating a decreased ReHo in patients with GHD, *BA* Brodmann's area, *L* left, *R* right, *PUT* putamen, *PreCG* precentral gyrus, *ORBsup* superior frontal gyrus, orbital part, *ORBsupmed* superior frontal gyrus, medial orbital, *SFGmed* superior frontal gyrus, medial.

## Discussion

To the best of our knowledge, this is the first study to analyze the regional homogeneity of brain activity in children with GHD in a resting state. In the current study, we performed rs-fMRI and ReHo analysis in children with GHD and HCs. Compared to HCs, children with GHD had increased ReHo in the left PUT, and decreased ReHo in the right PreCG, bilateral ORBsup and left SFGmed. These findings indicate that children with GHD have altered brain activity compared to HCs.

Compared with the HCs, the GHD group showed significantly increased ReHo in the left PUT. It has been suggested that this brain region plays a vital role in inhibitory control^25^. Several studies have found that the PUT is more active in more impulsive subjects than in less impulsive subjects^26–29^. Furthermore, in recent fMRI studies, motivated behavior has been shown to be accompanied by a weakened cognitive control of impulses^30^. Consistent with a prior study on GHD^16^, we also found significantly decreased ReHo in the right PreCG in children with GHD. The PreCG is located in the premotor cortex and is associated with the somatic sensorimotor network^31–33^. Rech et al. found that the white matter underneath the premotor cortex could immediately elicit a unilateral negative motor response (UNMR) when stimulated^34^. Meanwhile, Schucht et al. suggested that the neural network causing UNMRs may be related to motor control^35^. Thus, we speculate that the GHD group’s increased ReHo in the left PUT and decreased ReHo in the right PreCG may be associated with motor control dysfunction, which may reflect the pathophysiological mechanism of impulsive and hyperactive characteristics of behavior in children with GHD^5,36,37^.

In the current study, we also observed decreased ReHo in children with GHD in bilateral ORBsup, a region that is located in the medial orbitofrontal cortex (mOFC). The ORBsup has been shown to play a vital role in choosing the right response and suppressing the wrong response^38,39^. In a previous study, patients with ORBsup lesions were found to have behavioral regulation failures in the absence of external stimuli^38^. Decreased mOFC activity can be detected when patients with anxiety and depression fail to inhibit inappropriate anxiety responses^40–42^. In addition, decreased ReHo was also observed in the SFGmed, located in the medial prefrontal cortex (MPFC). The MPFC is one of the most important functional areas in the default mode network^43^, and is considered to participate in emotional processing, such as monitoring anxiety and other psychological states^44–47^. The significantly decreased ReHo in the MPFC of anxiety patients was linked to a decrease in the ability to regulate emotion^13^. In the present study, the decreased ReHo of the ORBsup and SFGmed in the GHD group may be associated with inactivation of emotional regulation, which may underlie the anxiety and depression observed in children with GHD^5,36,37,41^.

Several limitations of this study need to be addressed. Firstly, the number of subjects included in the study was small, especially in the HC group. Therefore, the generalizability of this study to the whole GHD population is limited. However, since both gender and age were matched between the two groups, we believe this constraint did not affect the robustness of our results. Secondly, participants in this study were not assessed by clinical cognitive and behavioral scales. It is valuable to evaluate the relationship of neuroimaging results with clinical scales. Therefore, in future studies, assessments of relevant scales should be included.

## Methods

### Participants and ethical considerations

All subjects were recruited from the pediatric clinic of the Zhejiang Provincial People’s Hospital from October 2016 to March 2018. All participants’ parents gave written informed consent for their inclusion in the study, and this study was approved by the Medical Ethics Committee of Zhejiang Provincial People's Hospital and adhered to the tenets of the Declaration of Helsinki. All subjects were right-handed and prepubescent. Participants were excluded if they met the following criteria: (1) head movements were obviously too large during MRI scans; (2) abnormalities identified on MRI associated with GHD; (3) had congenital multiple pituitary hormone deficiency (MPHD) or thyroid axis hormone level abnormalities; (4) chronic liver, kidney, or skeletal system diseases; (5) chromosomal abnormalities or special facial/abnormal signs with diagnostic significance; (6) hyperthermia during the scanning; (7) past medical history of seizures, benign masses or cancers, or psychological disease/psychosis; (8) family history of mental retardation or congenital heart disease, or (9) contraindications to MRI (such as metal implants or claustrophobia).

Growth hormone (GH) stimulation tests were performed in participants with short stature. Because of the limitations of GH stimulation tests, GHD can only be diagnosed when the results of two or more drug stimulation tests are abnormal^1,48^. Seven children with short stature were excluded due to their peak GH value, which was higher than 10 μg/L, during the GH stimulation test. Accordingly, the first group was composed of 26 children with GHD (11 males, mean age: 8.2 ± 1.9 years), in whom both arginine and clonidine GH stimulation tests showed a partial or complete lack of GH (< l0 μg/L)^1^. Furthermore, 15 age- and sex-matched HCs formed the second group (7 males, mean age: 8.6 ± 1.7 years), who had normal height and normal growth variation. There were no statistically significant differences in age (*p* = 0.3823, two-tailed Mann–Whitney *U* test) or sex (*p* > 0.9999, two-tailed Fisher’s exact test) between the two groups.

### Imaging data acquisition

fMRI scans were performed with a 3.0-T MRI scanner (Discovery MR750; GE Healthcare, Milwaukee, WI) prior to GH stimulation tests in all participants during the resting state to avoid the effects of stimulating drugs. The subjects were instructed to lie as still as possible but not fall asleep. Two foam pads were used on the head to reduce head movement, and earplugs were worn to reduce external noise. The MRI equipment and data parameters used in all MR imaging examinations were consistent with our previous study^49^. The specific parameter settings were as follows: repetition time = 2000 ms, echo time = 30 ms, flip angle = 90°, field of view = 220 mm, matrix size = 64 × 64, voxel size = 3.44 × 3.44 × 3.2 mm^3^, no slice gap and 44 axial slices. The first 10 volumes of all acquired rs-fMRI data image sequences were excluded in order to ensure that the subjects were stable in the resting state.

### Data preprocessing

The rs-fMRI data processing was performed using DPABI software (Version V4.0_190305, http://rfmri.org/dpabi)^50^, followed by temporal difference and head-motion corrections on the remaining 200 volumes. No participants were excluded for translation or rotation parameters exceeding 3.0 mm or 3.0° of head motion. Previous studies have reported that rs-fMRI findings is sensitive to head motion effects^51,52^. Here, we also computed the frame-wise displacement (FD) index^51^, and found no significant differences (*p* = 0.7175) in head movement between children with GHD (0.0936 ± 0.0632) and HCs (0.1009 ± 0.0584) using two-sample *t*-tests. The echo-planar imaging (EPI) template was used to spatially normalize the image into the Montreal Neurological Institute (MNI) space and resampled to 3 × 3 × 3 mm^3^ voxels. Subsequently, to remove the effects of head motion and other possible sources of artifacts, we regressed the constant, linear trend, and head motion parameters (Friston 24-parameter model)^53^ as covariates^54^. The time series signals of the cerebrospinal fluid and white matter were eliminated by using the prior template in SPM8 software (Statistical Parametric Mapping 8, http://www.fil.ion.ucl.ac.uk/spm/). Finally, a band-pass filter (0.01 < *f* < 0.08 Hz) was used to remove the effects of noise, such as respiratory rhythm.

### ReHo analysis

For the preprocessed rs-fMRI data, the time series of specific voxels were extracted. We defined the 27 nearest voxels as a cluster and calculated their ReHo. The Kendall’s coefficient concordance (KCC) values measure the consistency of activities between adjacent voxels and are assigned to a given voxel^17^, according to the following Eq. (1):

1 $$W = \frac{{\sum {\left( {R_{i} } \right)^{2} - n\left( {\overline{R}} \right)^{2} } }}{{\frac{1}{12}K^{2} \left( {n^{3} - n} \right)}}$$

where *W* was the KCC among given voxels, ranging from 0 to 1; *R*_*i*_ was the sum rank of the *i*th time point; $\overline{R} = \left( {\left( {n + 1} \right)K} \right)/2$ was the mean of the *R*_*i*_^’^s; *K* was the number of time series within a measured cluster (here *K* = 27); and *n* was the number of ranks (here *n* = 200). The individual KCC map was generated with the free DPABI software (Version V4.0_190305, http://rfmri.org/dpabi)^50^. Finally, spatial smoothing was performed in KCC maps with a full width at half maximum (FWHM) of 4 mm to decrease spatial noise.

### Statistical analysis

Second-level analyses of KCC maps were carried out using the SPM 8 software package. The brain regions with a KCC value greater than 1 were detected by performing one-sample t-tests within the two groups. A mask was used, according to the automated anatomical labeling (AAL) templates^55^, which matched the size of the spatially normalized voxels (3 × 3 × 3 mm^3^). The multiple comparisons were performed using the false discovery rate (FDR) criterion (*p* < 0.05)^56^.

The one-sample *t*-tests results from the two groups were combined to get a new map. By binarizing the map, a combined explicit mask was obtained. Then, two-sample *t*-tests were performed within this mask to compare the ReHo between two groups. The results of the two-sample *t*-tests were corrected by using the AlphaSim program in the RESTplus software (Resting-State fMRI Data Analysis Toolkit plus V1.24, http://www.restfmri.net/forum/RESTplus)^24^, as determined by a Monte Carlo simulation to calculate the probability of false-positive detection^13^. The AlphaSim correction was confined within the combined mask of one-sample t-tests results from the two groups, and the size of smooth kernel was set at 4 mm. The threshold of the *t*-map was set at a combined height threshold of *p* < 0.05 and a minimum cluster size of 56 voxels, and this corresponded to a corrected *p* < 0.05.

## Conclusions

In the current study, we performed fMRI and ReHo calculations in children with GHD and HCs in the resting state. Our findings of increased ReHo in the left PUT and decreased ReHo in the right PreCG may suggest a dysfunction of inhibitory control in children with GHD. In addition, the decreased ReHo was observed in the bilateral ORBsup and left SFGmed, reflecting a possible inactivation of the inhibition of inappropriate anxiety and depression in children with GHD. In summary, the present findings confirmed that the activity homogeneity in specific brain regions is altered in children with GHD, providing imaging evidence to elucidate potential pathophysiological mechanisms of abnormal cognition and behavior in children with GHD.
